# Knockout of Zebrafish Ovarian Aromatase Gene (*cyp19a1a*) by TALEN and CRISPR/Cas9 Leads to All-male Offspring Due to Failed Ovarian Differentiation

**DOI:** 10.1038/srep37357

**Published:** 2016-11-23

**Authors:** Esther Shuk-Wa Lau, Zhiwei Zhang, Mingming Qin, Wei Ge

**Affiliations:** 1 School of Life Sciences, The Chinese University of Hong Kong, Shatin, New Territories, Hong Kong, China; 2 Centre of Reproduction, Development and Aging (CRDA), Faculty of Health Sciences, University of Macau, Taipa, Macau, China

## Abstract

Sexual or gonadal differentiation is a complex event and its mechanism remains elusive in teleosts. Despite its complexity and plasticity, the process of ovarian differentiation is believed to involve gonadal aromatase (*cyp19a1a*) in nearly all species studied. However, most data concerning the role of aromatase have come from gene expression analysis or studies involving pharmacological approaches. There has been a lack of genetic evidence for the importance of aromatase in gonadal differentiation, especially the timing when the enzyme starts to exert its effect. This is due to the lack of appropriate loss-of-function approaches in fish models for studying gene functions. This situation has changed recently with the development of genome editing technologies, namely TALEN and CRISPR/Cas9. Using both TALEN and CRISPR/Cas9, we successfully established three mutant zebrafish lines lacking the ovarian aromatase. As expected, all mutant fish were males, supporting the view that aromatase plays a critical role in directing ovarian differentiation and development. Further analysis showed that the ovarian aromatase did not seem to affect the formation of so-called juvenile ovary and oocyte-like germ cells; however, it was essential for further differentiation of the juvenile ovary into the true ovary.

In fish, the most common types of sexuality are hermaphroditism with male and female gonads present at the same time and gonochorism with only one type of gonad in each individual. Most gonochoristic fish lack dimorphic sex chromosomes and their sexuality is determined at early developmental stage when gonads start to differentiate. Unlike in mammals, gonadal differentiation in teleost fish shows greater plasticity^1^,^2^, which is influenced by a variety of factors including genetic background (monogenic or polygenic), environmental condition (*e*.*g*., temperature, density, pH, and hypoxia), social interaction (*e*.*g*., social dominance) and endocrine factors (*e*.*g*., sex steroids)^3^,^4^,^5^,^6^.

Gonadal steroids are well known to play important roles in fish gonadal development (gonadogenesis) and function. One of the critical genes of steroidogenesis is aromatase (CYP19), which converts androgens to estrogens^7^. Estrogens are well known to be important in promoting ovarian development and maintaining femaleness afterwards^4^,^8^. Unlike the aromatase in mammals, fish aromatase is coded by two separate genes, the gonad-specific *cyp19a1a* and brain-specific *cyp19a1b*^9^,^10^,^11^. The gonad-specific *cyp19a1a* is primarily expressed in the ovary, mostly in the granulosa cells of the follicle for the synthesis of estrogens^12^,^13^. The importance of *cyp19a1a* in sexual or gonadal differentiation has been well studied in teleosts, focusing mostly on its expression and regulation during gonadal differentiation and the response of differentiating gonads to the administration of estrogens or estrogenic substances^8^. The activity of aromatase and/or its expression during gonadal differentiation has been demonstrated in a variety of species including rainbow trout^14^, tilapia^14^,^15^, ricefield eel^16^, and European seabass^17^. The importance of aromatase in gonadal differentiation has also been shown by inhibiting aromatase enzymatic activity with aromatase inhibitors, which induced sex reversal in fish from genetic females to phenotypic males^14^,^18^,^19^. Similar change has also been induced by blocking estrogen signaling with estrogen receptor antagonists^20^. In addition, treatment of tilapia with masculinizing temperature caused a down-regulation of *cyp19a1a*^3^,^21^. There was evidence that the temperature-induced decrease in *cyp19a1a* expression might be due to, at least partially, a suppression of follicle-stimulating hormone receptor (*fshr*) expression in the Japanese flounder^22^.

Zebrafish is a gonochoristic species at adult stage; however, it is also considered a juvenile hermaphrodite because the fish first develops ovary-like gonads or juvenile ovaries, which are characterized by initiation of meiosis around 10 dpf followed by development of perinucleolar oocytes (POs) and ovarian cavity^23^. The development of testis therefore involves degeneration of the exiting oocyte-like germ cells through apoptosis followed by transformation of the gonad to testis^24^,^25^,^26^,^27^. This view is generally cited in the literature although it has been challenged by some studies because not all males seem to undergo a phase of juvenile ovary with typical POs^26^,^28^,^29^. Like many other fish species, the sexual or gonadal differentiation in the zebrafish is highly plastic and also influenced by both internal (genes and hormones)^27^,^30^,^31^,^32^ and external factors (*e*.*g*., temperature and hypoxia)^33^,^34^. The expression of *cyp19a1a* could be detected during early development including the periods before and when gonadal differentiation occurs^35^,^36^. A decrease in *cyp19a1a* expression during sex differentiation period was associated with disappearance of the juvenile ovary and appearance of germ and somatic cells that eventually developed into testicular tissues^24^. Exposure to estrogens or estrogenic substances during zebrafish development significantly altered sex ratio towards female dominance with some individuals having ovotestes^30^. Treatment of zebrafish with aromatase inhibitor during gonadal differentiation resulted in 100% masculinization^37^. A recent study showed that sexually mature female zebrafish could also be induced to undergo sex reversal by treatment with aromatase inhibitor^38^. High temperature can significantly influence sex ratio in the zebrafish. This effect also seemed to be mediated by inhibiting *cyp19a1a* gene expression, leading to oocyte apoptosis and development of testicular tissue during sex differentiation^39^. Environmental hypoxia has been reported to cause male-dominant population in the zebrafish, and again it is associated with a decreased expression of *cyp19a1a*^40^.

All these lines of evidence from physiological, pharmacological and environmental studies indicate that aromatase is a key factor in driving ovarian differentiation in fish including the zebrafish. This is in contrast to findings in the mouse. The experiments using aromatase or estrogen receptor knockout mice have shown that estrogen is essential for normal ovarian function, but not for sex differentiation or ovarian formation^41^,^42^. The female aromatase knockout mice are infertile due to a blockade of follicular development at the antral stage, resulting in disrupted folliculogenesis and failed oocyte maturation and ovulation^43^,^44^. The knockout approach, however, had not been available in fish species including the zebrafish due to the lack of relevant technologies in non-rodent models.

Recently, two efficient and powerful genome-editing technologies, Transcription Activator-Like Effector Nuclease (TALEN)^45^,^46^ and Clustered Regularly Interspaced Short Palindromic Repeats/CRISPR-associated system (CRISPR/Cas9)^47^,^48^, have emerged, allowing disruption of any genes of interest in various species including the zebrafish. Using different mechanisms, TALEN and CRISPR can both induce double-strand breaks (DSB) in the genome, resulting in insertion and/or deletion (Indel mutation) in the genome after non-homologous end-joining (NHEJ) or specific mutation after homologous recombination (HR)^49^. TALEN involves two TALE proteins each carrying a FokI nuclease. After binding to the target sites with a spacer of 15–20 bp in between, the FokI nucleases dimerize and cut the DNA at the target site^49^. On the other hand, the CRISPR/Cas9 method relies on a synthetic single guide RNA (sgRNA) to recognize the target site and direct the Cas9 nuclease to the target^50^. CRISPR/Cas9 is simpler and easier to construct than TALEN; however, due to the short recognition site of only 18 nucleotides, off-targeting effect may occur^51^.

With these convenient and powerful genetic tools, zebrafish has become one of the top models for investigating functions of interested genes *in vivo* using gene knockout approach, which has been restricted mostly to the mouse model. Using TALEN, we have recently analyzed the functions of pituitary gonadotropins (FSH and LH) and their receptors (Fshr and Lhcgr) in the zebrafish in both females and males^52^,^53^. Using the same approach, we have undertaken the present study to evaluate the role and importance of ovarian aromatase (*cyp19a1a*) in zebrafish gonadal differentiation. In addition to TALEN, we also adopted CRISPR/Cas9 method, which allowed us to compare the effectiveness of the two methods on the same gene. Three null mutant zebrafish lines were established using TALEN and CRISPR/Cas9 respectively for phenotype analysis on sex ratio and gonadal development.

## Results

### Establishment of cyp19a1a mutant lines by TALEN and CRISPR/Cas9

To disrupt ovarian aromatase gene (*cyp19a1a*) in the zebrafish, we first adopted TALEN, which we have used successfully to mutate genes of gonadotropins (*fshb* and *lhb*)^52^ and their receptors (*fshr* and *lhcgr*)^53^. We first targeted four sites at the exon 1 and 3; unfortunately, none of these sites worked for unknown reasons. We then tried the fifth TALEN site at exon 2 and 2 CRISPR/Cas9 sites at exon 1 and 2 respectively (Supplementary Fig. 1). The fifth TALEN site worked, so did both CRISPR sites. We therefore created three independent mutant lines [1 from TALEN (−5) and 2 from CRISPR (−5; −3, +12)] for phenotype analysis.

For TALEN knockout, we used the plasmids pCS2TAL3DD and pCS2TAL3RR reported previously^46^. To increase the efficiency of mutant screening, we modified the plasmids by inserting an EGFP and mCherry gene into the left and right TALE protein respectively. The injected embryos were screened under a fluorescent or sometimes confocal microscope for TALE protein expression (Supplementary Fig. 2). Our result showed that the embryos showing strong fluorescent signals exhibited significantly higher mutation rate (data not shown). By eliminating the embryos with no or weak signals, we could efficiently enrich those with mutations.

**Figure 1:**
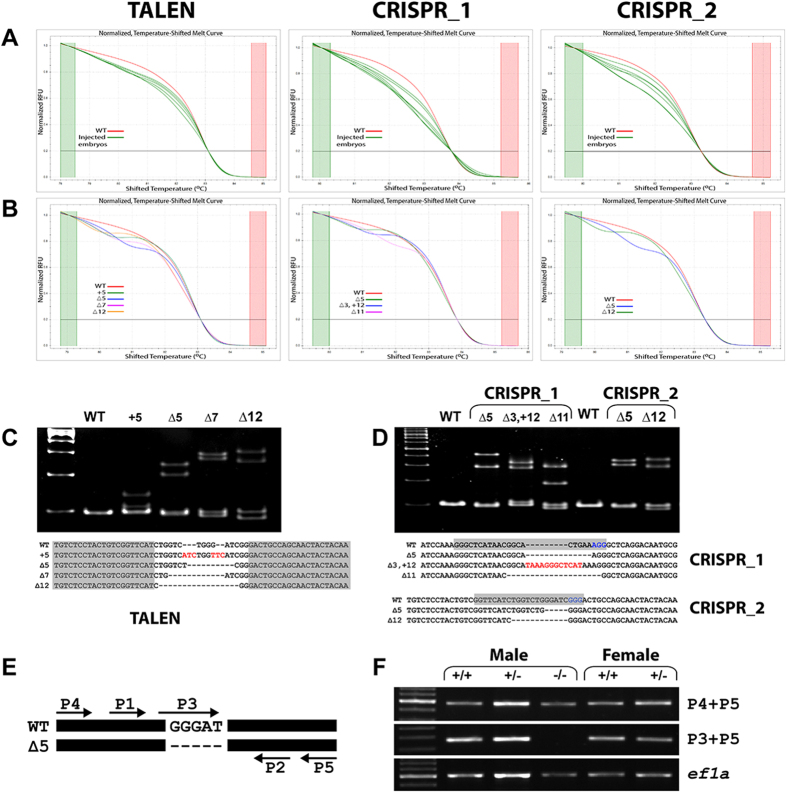
Genotype analysis of *cyp19a1a* mutants. (**A**) Mosaic F0 embryos showing shifted HRMA curves (green) with the uninjected control ones (red). (**B**) HRMA curves of the heterozygous F1 as compared with that of the WT individuals (red). (**C** and **D**) The representatives of TALEN and CRISPR-induced germline mutations. Sequences in shadow indicate the TALEN binding sites or CRISPR target sites. The sequences highlighted in blue are PAM sites for CRISPR targeting and those in red are inserted sequences. The gel images on the top are HMA assays on the representative mutants. Each lane is corresponding to a mutant sequence listed below the gel image. (**E**) Schematic illustration of the primers used for PCR demonstration of mutation. The primer P3 is specific to the mutated site. (**F**) PCR verification of mutant DNA with primers P3 and P5. *Ef1a* is the housekeeping gene as the control. The full-length gels are included in supplementary information.

Both TALEN and CRISPR/Cas9 approaches were able to generate indel mutations in *cyp19a1a* gene (HRMA, Fig. 1A and B; HMA, Fig. 1C and D), and we did not observe significant difference between the two approaches in terms of efficiency (data not shown). The loss of *cyp19a1a* gene was also confirmed by semi-quantitative PCR detection by using a mutant-specific primer (P3) (Fig. 1E and F).

### All-male development in cyp19a1a-deficient fish

**Figure 2:**
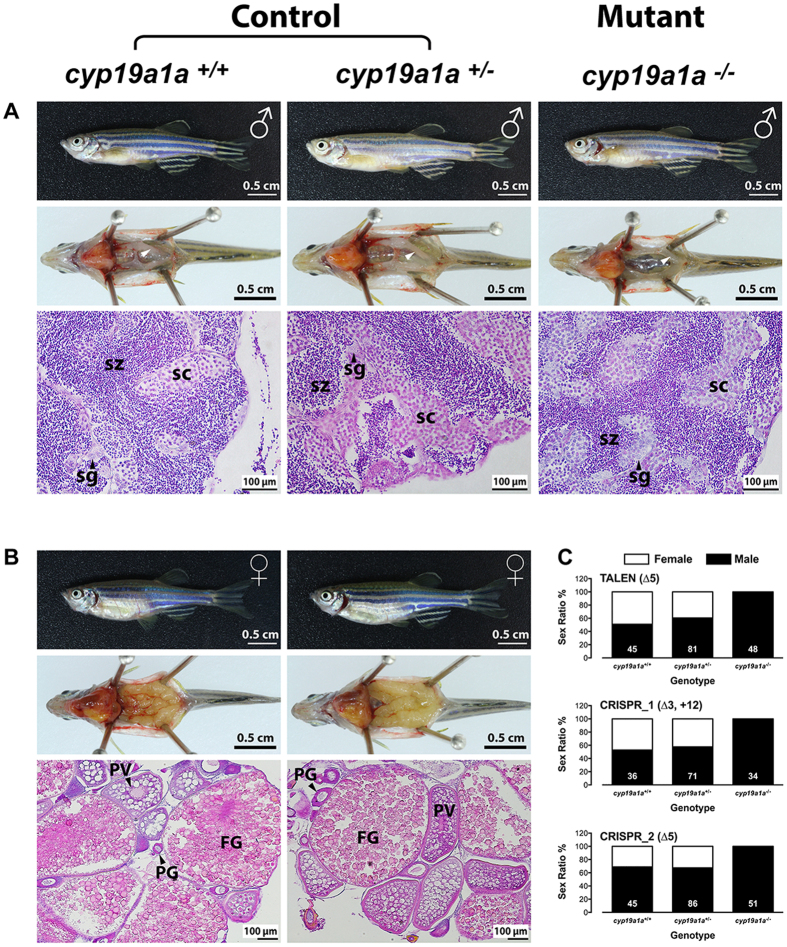
All-male development in *cyp19a1a* mutants. (**A** and **B**) Anatomical and histological examination of the gonads in the control (*cyp19a1a*^+/+^ and *cyp19a1a*^+/−^) and mutant (*cyp19a1a*^*−/−*^). (**C**) Sex ratio of the three *cyp19a1a* mutants. The number in each column indicates the sample size. sg, spermatogonia; sc, spermatocytes; sz, spermatozoa; PG, primary growth follicle; PV, previtellogenic follicle; FG, full-grown follicle.

Aromatase is well known to be involved in sex differentiation in teleosts. To confirm this function, we first examined the sex ratio of all three mutant zebrafish lines around 120 dpf (zebrafish becomes sexually mature at 60–90 dpf in our aquarium system). As expected, all the mutant individuals examined were males, while the wild type (+/+) and heterozygous (+/−) controls for each mutant had both males and females (Fig. 2A and B) with similar sex ratios (Fig. 2C). Histological examination of the TALEN mutant showed that all genotypes (+/+, +/−, and −/−) exhibited normal spermatogenesis in the testis with no observable difference (Fig. 2A). The males of the mutant fish (−/−) were fertile as tested by crossing with the wild type control females (data not shown).

### Real-time monitoring of gonadal differentiation

All-male phenotype at adult stage could be due to either failed function of the signaling pathways leading to ovarian development during gonadal differentiation or sex reversal from female to male after ovarian formation as we recently observed in *fshr* mutant^53^. According to the studies reported so far^23^,^25^, the testis development in the zebrafish, at least in some individuals, involves apoptosis of oocyte-like germ cells and transformation from juvenile ovary to testis^24^,^26^. The process is so fast and transient that it is often difficult to reveal by random sampling for histological analysis.

To monitor gonadal differentiation *in vivo* without sacrificing the fish, we crossed the *cyp19a1a* mutant created with TALEN with the transgenic fish line *Tg*(*vas:EGFP*), which expresses EGFP in the germ cells under germ cell-specific *vasa* promoter^54^. Vasa is an ATP-dependent RNA helicase specifically expressed in the germ cells and it has been used as a germ cell-specific marker to label and visualize the germ cells during gonadal development^26^,^55^. This particular transgenic line, however, was reported to expresses GFP abundantly in the ovary but much less in the testis^26^,^56^. The signal intensity of *vasa* promoter-driven GFP decreases in the process of juvenile ovary-to-testis transformation while increases in the ovary^26^. This sexually dimorphic expression of GFP makes this transgenic line a useful model to monitor gonadal differentiation *in vivo*.

**Figure 3:**
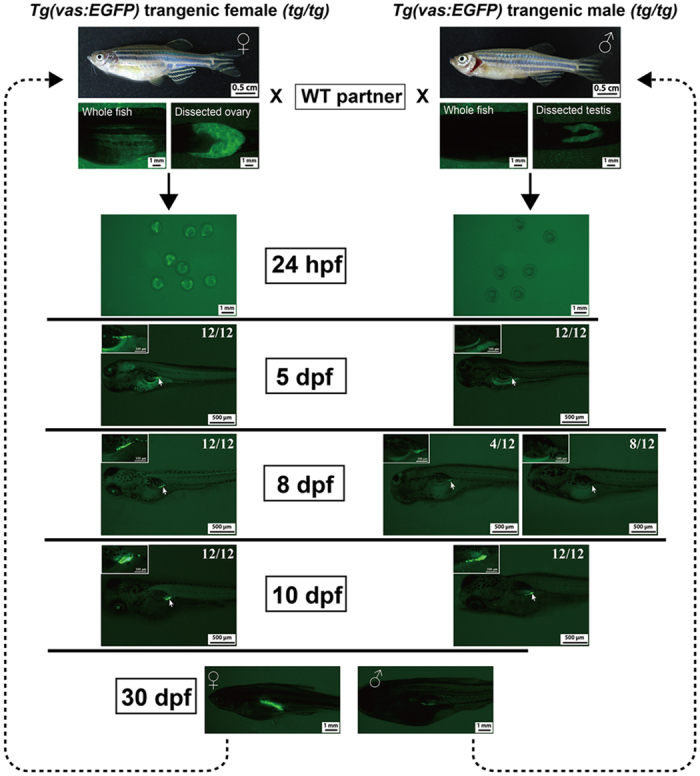
Expression of GFP in the gonads of the transgenic line *Tg*(*vas:EGFP*). The embryos/offspring were obtained by crossing the WT fish with either female (*tg*/*tg*) or male (*tg*/*tg*) transgenic fish. The GFP signal was observed under fluorescent microscope from 24 hpf to 30 dpf (12 individuals each time).

By introducing the *vas:EGFP* reporter to the aromatase mutant, we were able to trace the early gonadal development in the mutant fish [*Tg*(*vas:EGFP*)*;cyp19a1a*^−/−^], allowing accurate sampling for histological analysis. As shown in Fig. 3, the female adult transgenic fish (*tg/tg*) showed strong GFP signal in the ovary under fluorescent stereomicroscope. In contrast, the testis from the transgenic male showed much weaker GFP signal, which could barely be seen through the body wall without dissection. This feature is useful for gender identification, especially before sexual maturation. As reported previously^54^, we observed that the heterozygous offspring (*tg/w*) from homozygous transgenic female and wild type male showed GFP signal in the germ cells throughout embryonic and post-hatching development including primordial germ cells (PGCs), probably due to the maternal mRNA specifically localized in the germ cells; however, those from homozygous transgenic male and wild type female had no signal in the germ cells until 8 dpf when some individuals (4 out of 12; 4/12) started to show signal in the gonads. This is likely due to the lack of an essential element in the transgene promoter for expression in the embryonic germ cells^54^. Our observation differed from the previous reports that the zygotic expression of the transgene GFP started in most of the individuals around 16–24 dpf 26 or 21–23 dpf 56, respectively. At 10 dpf, all juveniles from either crossing combination exhibited visible GFP signal in the undifferentiated gonads with equal signal intensity. This lasted until gonadal differentiation around 25 dpf when the GFP signal became intensified in some fish that were destined to become females; however, in those that later developed into males, the signal remained unchanged or even decreased (Fig. 3). The timing of GFP signal divergence agreed well with that reported by Krovel and Olsen^56^. This provides a useful marker for assessing the onset and extent of gonadal differentiation.

### Role of ovarian aromatase in sex differentiation

Although it was expected that the lack of aromatase would lead to all-male individuals, the exact stage of development at which the enzyme works to influence gonadal differentiation remained unknown. To address this issue, we examined the juvenile fish from 20 to 47 dpf, covering the period from gonadal differentiation to female puberty onset in the zebrafish^52^,^57^.

**Figure 4:**
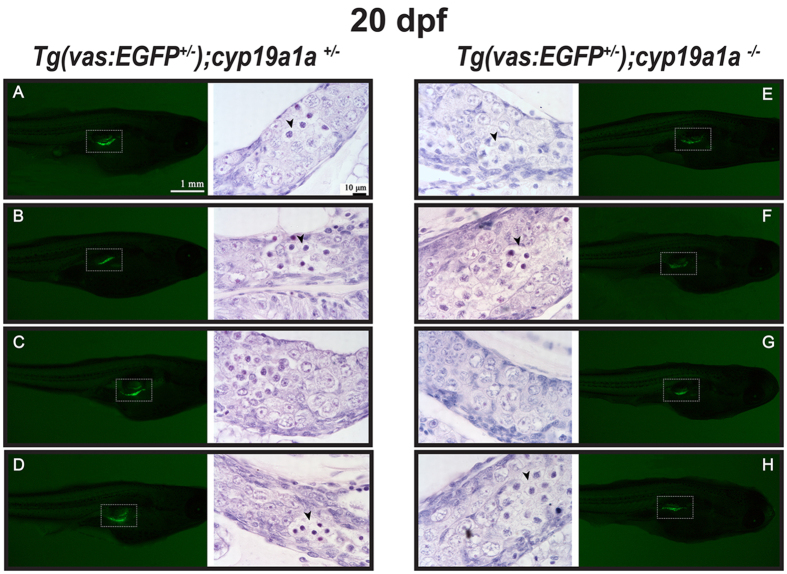
Gonad development at 20 dpf in the control [*Tg*(*vas:EGFP*)*;cyp19a1a*^+/−^; fish **A**–**D**] and mutant [*Tg*(*vas:EGFP*)*;cyp19a1a*^−/−^; fish **E**–**H**]. Similar GFP signals (boxed in the photo) were observed in the two groups and histological examination showed no significant difference. Meiotic germ cells (arrowhead) with condensed chromatin were often seen in both the mutant and control gonads.

GFP signals were observed in both *cyp19a1a* mutant fish (−/−) and their sibling control (+/−) at 20 dpf, and individuals of the same genotype showed similar GFP intensity (Fig. 4). Interestingly, the GFP signal in the mutant fish (Fig. 4E–H) seemed to be slightly weaker than that of the control (Fig. 4A–D). Despite this, histological analysis showed little difference in gonadal development between the two genotypes. Although the meiotic cells were abundant in both genotypes, the gonocytes or gonial germ cells in both the control and mutant gonads were basically undifferentiated at the histological level with no obvious signs of sex differentiation (Fig. 4). Since we could not distinguish male and female germ cells based on morphological features, we would consider the gonads at this stage indifferent as suggested previously^25^ rather than ovary-like although the entry of the germ cells into meiosis suggests that the gonads might have committed femaleness at molecular level^23^. Although the term oocyte-like germ cell is frequently used in the literature, very few studies have clearly defined their characteristics^24^,^25^,^26^,^58^. In this study, we consider the germ cells to be oocyte-like only when they exhibited typical perinucleolar features with diameters being 20 μm or bigger^59^ and with basophilic cytoplasm and prominent nucleoli located at the periphery of the nuclei^25^.

**Figure 5:**
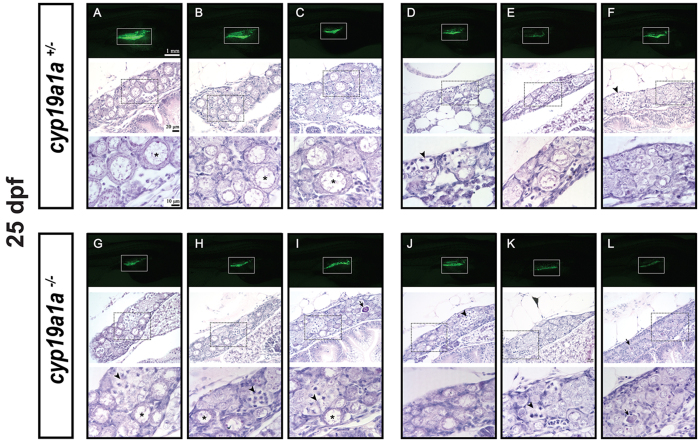
Gonad development at 25 dpf in the control (*cyp19a1a*^+/−^; fish **A**–**F**) and mutant (*cyp19a1a*^−/−^; fish **G**–**L**). The GFP intensity in the gonads (boxed in the photo) started to diverge in the control (**A**–**C** vs. **D**–**F**) but not the mutant fish (**G**–**L**), suggesting the start of gonadal differentiation. Two groups of fish could be distinguished by histological analysis. One group had well-developed EPOs (asterisks) in the gonads (fish **A**–**C**) in the control and (**G**–**I**) in the mutant) and the other contained mostly undifferentiated germ cells (fish **D**–**F**) in the control and (**J**–**L**) in the mutant. The EPOs in the control fish (**A**–**C**) were generally larger with stronger GFP signal than those in the mutant fish (**G**–**I**). Numerous meiotic germ cells (arrowheads) were present in the gonads of both control and mutant fish. The dark-stained condensed apoptotic germ cells (arrows) were often observed at this stage, indicating the process of juvenile ovary-testis transformation.

At 25 dpf, the gonadal GFP signals started to diverge among individuals in the control fish, which could be divided into two groups with one having significantly increased GFP signal. Histological analysis showed that the fish with enhanced GFP signal all had typical perinucleolar oocytes (PO; primary growth stage, PG) in the gonads (Fig. 5A–C), whereas those with similar or reduced GFP signal had small oocyte-like early POs (EPOs) or undifferentiated germ cells (Fig. 5D–F). In the mutant fish without aromatase, the GFP signal in all individuals remained more or less the same. In some mutant fish, the gonads contained EPOs, which were obviously smaller in size than those in the control fish (Fig. 5G–I). Both meiotic and apoptotic germ cells were commonly seen in the mutant gonads at this stage of development. In some individuals of both control and mutant groups, the gonads had increased amount of stromal cells among germ cells or germ cell cysts, a sign of testis development in genetically male fish^25^.

**Figure 6:**
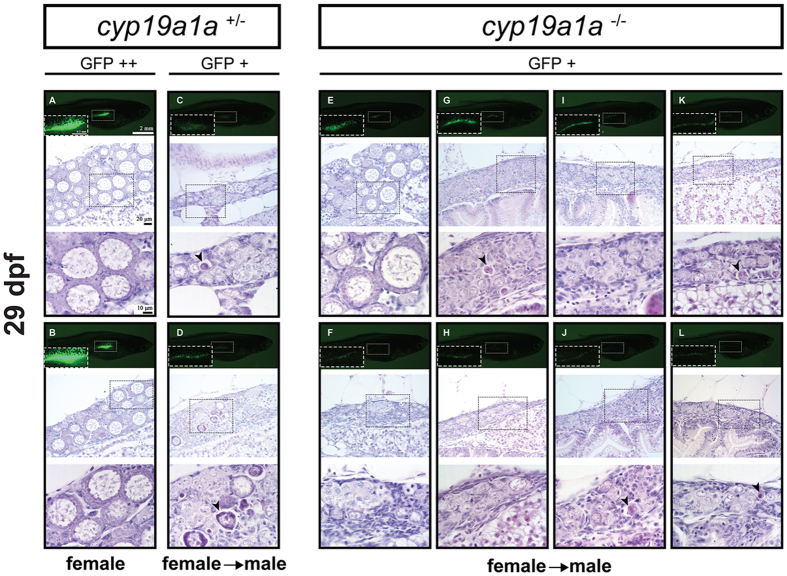
Gonad development at 29 dpf in the control (*cyp19a1a*^+/−^; fish **A–D**) and mutant (*cyp19a1a*^−/−^; fish **E**–**L**). In the control fish, the gonads (boxed in the photo) with much enhanced GFP signal (GFP++; **A** and **B**) contained large LPOs and little amount of stromal tissues, indicating the development towards the true ovary whereas those with weak or reduced GFP signal (GFP+; **C** and **D**) had few POs but large number of apoptotic cells (arrowheads), suggesting the development towards males. In the mutant fish, all the mutant individuals showed weak GFP signal comparable with that of the control males. Most mutant gonads (**G**–**L**) contained undifferentiated germ cells with some undergoing apoptosis (arrowheads). A few contained OPs (*e*.*g*., fish **E**) but with large amount of stromal tissues.

At 29 dpf, the GFP signal continued to show dynamic changes. In the control fish (*cyp19a1a*^+/−^) (Fig. 6A–D), some individuals showed strong GFP signal (GFP++; Fig. 6A and B) in the gonads, and histological analysis confirmed that all these fish had well-developed ovaries with late perinucleolar oocytes (LPO). By comparison, the control fish with weak GFP signal (GFP+; Fig. 6C and D) were undergoing transformation to become testis, which was characterized by high numbers of apoptotic germ cells as well as high amount of stromal cells among germ cells (Fig. 6C and D). In contrast, all mutant fish (*cyp19a1a*^−/−^) showed weak GFP signals, similar to the male control fish, and in some individuals the signal became too weak to observe. Histological examination demonstrated that most (7/8) of the mutants (Fig. 6F–L) showed indifferent gonads with large number of apoptotic germ cells and high amount of stromal tissues, suggesting that these gonads were undergoing the juvenile ovary-to-testis transformation although no male-specific cystic structure with spermatocytes was visible at this stage. In rare individuals such as the one in Fig. 6E, we could see LPOs or PG follicles, but the interfollicular space was filled with stromal cells.

**Figure 7:**
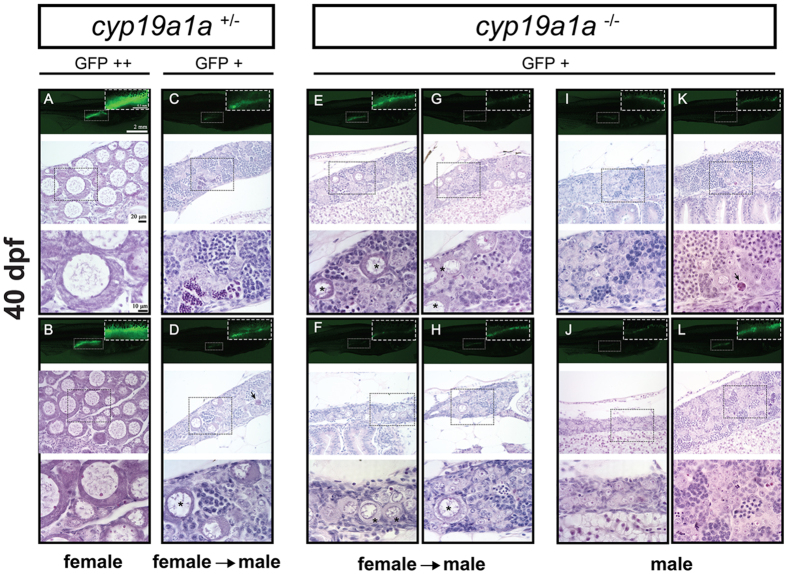
Gonad development at 40 dpf in the control (*cyp19a1a*^+/−^; fish **A**–**D**) and mutant (*cyp19a1a*^−/−^; fish **E**–**L**). The control fish had well-differentiated ovary (**A** and **B**) and testis (**C** and **D**), whereas all the mutant individuals were undergoing or had completed ovary-testis transformation with typical testicular tissues containing different stages of spermatogenic cells. Some individuals (**E**–**H**) still contained a few typical EPOs (asterisks) scattered among the testicular tissues. Arrows indicate the apoptotic germ cells.

The typical testis structure (cystic spermatogenic cells) started to appear in the gonads at 40 dpf (could be earlier). In the control fish, the GFP signal continued to polarize with stronger signal in females (Fig. 7A and B) and weak signal in males (Fig. 7C and D). Most mutant fish had spermatogenic cells present in the gonads (Fig. 7E–L) with some still in the process of juvenile ovary-to-testis transformation as indicated by the presence of EPOs among the spermatogenic cysts (Fig. 7E–H).

**Figure 8:**
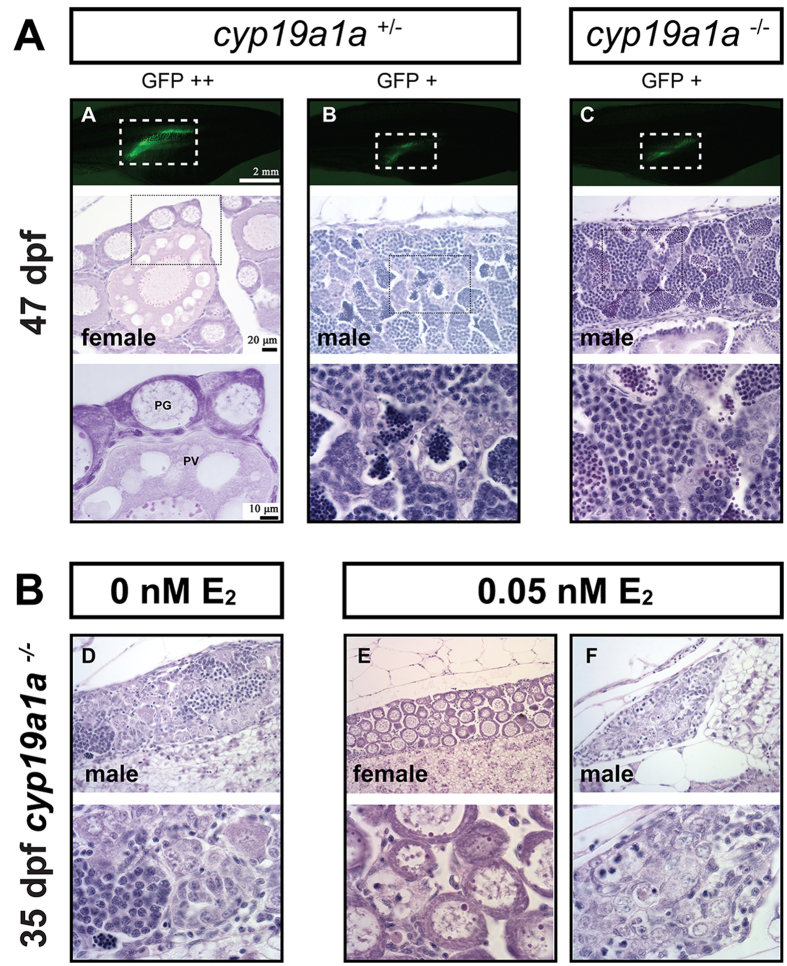
(**A**) Gonad development at 47 dpf in the control (*cyp19a1a*^+/−^) and mutant (*cyp19a1a*^−/−^). The gonadal differentiation had finished in the control fish with the ovary and testis well developed. The ovary contained both PG and PV follicles, indicating puberty onset in the female. All mutant fish were males with well-developed testes containing all stages of spermatogenic cells without any oocytes. (**B**) Rescue of mutant phenotype by E2 treatment. The juvenile mutant fish were treated with E2 (0.05 nM) from 15 to 30 dpf, and the fish were sampled for histological examination at 35 dpf. E2 treatment could induce normal ovarian formation in some mutant fish as shown by fish E. Other fish (*e*.*g*., fish F) still had undifferentiated gonads that were likely destined to males.

At 47 dpf, the gonads had completed the process of differentiation. Both females and males were present in the control group. The males had well-developed testis showing the entire process of spermatogenesis, whereas the females started to enter puberty as marked by the appearance of the previtellogenic follicles (PV, or stage II with cortical alveoli). No females were found in the aromatase mutant fish, and all individuals had well-developed testis without EPOs (Fig. 8A).

### Rescue of mutant phenotype by estradiol (E2) treatment

To provide further evidence for the role of estrogen in inducing ovarian differentiation, we performed an experiment to rescue the phenotype of *cyp19a1a* mutant by E2 treatment (0.05, 0.50 and 5.00 nM) over the time of gonadal differentiation (15–30 dpf). The result showed that exposure to E2 caused normal ovarian formation with fully developed POs and little amount of stromal tissues, and the effect could be observed in some individuals even at low concentration (0.05 nM) (Fig. 8B).

## Discussion

Sex determination and gonadal differentiation have been extensively studied in vertebrates^1^. Unlike the mammalian models, the mechanisms of these events show great diversity and plasticity in teleosts^1^,^60^, and no single mechanism has been found to work in most fish species studied, let alone all species. Despite the mechanistic diversity and developmental plasticity of gonadal differentiation, gonadal aromatase (*cyp19a1a*) is generally believed to play an important role in directing or influencing female development or ovarian differentiation^8^,^61^.

Although all the information accumulated points to the importance of aromatase in ovarian differentiation and development in fish models^8^, there is a lack of genetic data on its importance in the event, especially the timing of its action during gonadal differentiation. By using the powerful genome editing tools of both TALEN and CRISPR/Cas9, we disrupted the gene of gonad-specific aromatase *cyp19a1a* in the zebrafish. As expected, all three mutant lines we generated turned out to be all males, supporting the view that gonadal aromatase and the estrogens it produces are critical in directing gonadal differentiation. Interestingly, although aromatase has been reported to be expressed in the testis of both mammals^62^ and fish including the zebrafish^63^,^64^, implicating a role in spermatogenesis, we did not observe any abnormalities in spermatogenesis of the mutant fish, which were fertile as normal at 120 dpf. This agrees with the reports in the mouse. Despite some minor phenotypes such as enlarged male accessory glands, the aromatase knockout male mice exhibited normal fertility^43^. Interestingly, a separate study showed that although male mice deficient in aromatase were fertile initially, they gradually lost fertility at late stage with disruption of spermatogenesis in the testis^65^. Whether the male *cyp19a1a* mutant zebrafish displays any change of fertility over time remains unknown, and it will be an interesting issue to address in the future.

Zebrafish is considered a juvenile hermaphroditic fish whose gonad develops as juvenile ovary first with oocyte-like germ cells (meiotic and perinucleolar stage) followed by sexual differentiation afterwards at later stage. For genetic females, the juvenile ovaries develop further into true ovaries without tissue remodeling; however, the differentiation of testis involves a process of degeneration (apoptosis) of the oocyte-like germ cells including EPOs, which is accompanied by stromal tissue remodeling for differentiation into testicular tissues. This pattern of testis development was first proposed by Takahashi in 1977^23^ and has later been confirmed by a series of studies^24^,^25^. However, this view has also been questioned by studies using both morphological and genetic markers. Using the same transgenic zebrafish line as we used in the present study, Wang and his colleagues demonstrated that although the so-called juvenile ovaries might exist in the zebrafish at early stage, there was a significant variation in the degree of such female commitment with some individuals developing straight to males from indifferent gonads without going through an obvious stage of juvenile ovary^26^. However, if we consider the early entry into meiosis as a female-specific marker^66^,^67^, the widespread existence of meiotic cells in juvenile gonads would support the view that all individuals develop femaleness first (20–25 dpf) at cell and molecular levels with some individuals, but not all, showing significant morphological features of ovary such as perinucleolar oocyte (PO).

To provide evidence for the importance of aromatase and its timing of action during gonadal differentiation, we performed detailed histological analysis on gonadal development in control (*cyp19a1a*^+/−^) and mutant zebrafish (*cyp19a1a*^−/−^) from 20 dpf before differentiation starts to 47 dpf when puberty onset in females is expected^57^. Based on the change of GFP signal and histological analysis, we may conclude that there was no sexual differentiation at 20 dpf at morphological level; however, the meiotic cells were commonly seen in most individuals, indicating juvenile femaleness. The differentiation of the ovary and testis occurred around 25 dpf when the intensity of GFP signal started to increase in some control fish. Histological observation showed that these fish had well-developed EPOs in the gonads. Interestingly, some mutant fish also contained EPOs in the gonads, but their sizes were smaller than those in the control. Based on these observations, we propose that although the gonadal aromatase is expressed as early as 17 dpf 35, it is not involved in the formation of juvenile ovary as indicated by the meiotic germ cells and typical EPOs in the mutant ovary from 20 to 40 dpf. The aromatase, however, is important in directing or supporting further development of the juvenile ovary into true ovary and maintaining the femaleness afterwards. Without the support of the aromatase, the juvenile ovary cannot sustain the EPOs for long because a few days later (29 dpf) most mutant fish contained only undifferentiated gonocytes (presumably spermatogonia) in the gonads with many germ cells undergoing apoptosis (degenerating EPOs). Spermatogenesis became obvious at 40 dpf, and in some mutant fish typical EPOs could still be seen among testicular tissues. The importance of ovarian aromatase in driving ovarian differentiation and maintenance is also supported by a recent genetic study in tilapia, which showed that the knockdown of *cyp19a1a* in mosaic F0 fish by TALEN induced sex reversal in some individuals^68^.

The mutant phenotype could be successfully rescued by E2 treatment. Interestingly, E2 at low concentration (0.05 nM) could induce normal ovarian formation in some mutant individuals, but not all. This suggests that the dosage tested did not alter the male pathway, and that the ovarian differentiation in the zebrafish requires low concentrations of E2. The mutant zebrafish lines we established in this study will therefore provide a useful platform for testing estrogenic activities of endocrine disrupting chemicals.

In summary, using the powerful genome-editing tools, TALEN and CRISPR/Cas9, the present study provided the first genetic evidence for the importance of the gonadal aromatase in driving ovarian differentiation and development in fish. All three *cyp19a1a* mutant lines were males as expected. With the help of GFP transgene expression in the gonads, we carried out detailed histological examination of gonadal development and sexual differentiation. Our results suggest that the ovarian aromatase (*cyp19a1a*) plays a critical role in directing ovarian differentiation in the zebrafish. It does not seem to influence the formation of juvenile ovary with oocyte-like germ cells; however, it starts to function afterwards by promoting oocyte growth and maintaining their femaleness, which is essential for resisting germ cell apoptosis and further development into the true ovary.

## Methods

### Animals

The AB strain zebrafish obtained from CZRC (China Zebrafish Resource Center, Wuhan, China) was used for gene knockout in the present study. As reported previously^26^,^29^, we used a transgenic zebrafish line with EGFP expressed in the germ cells under *vasa* (now named *ddx4*, DEAD box polypeptide 4) promoter to monitor ontogenetic gonadal differentiation *in vivo*. The transgenic fish line *Tg*(*vas:EGFP*)^54^ was generously provided by Dr. Rüdiger Schulz (Utrecht University, The Netherlands). To obtain a stable line of AB background, we first crossed a male transgenic fish with an AB female followed by several rounds of backcrossing with the AB fish. The transgenic mutant fish was then created by crossing the *cyp19a1a* knockout mutant generated in the present study with the transgenic fish. All experiments were performed according to the guidelines and regulations set by the Research Ethics Committee of University of Macau and the Animal Experimentation Ethics Committee of The Chinese University of Hong Kong, and all experimental protocols were endorsed by both committees.

### Construction of EGFP and mCherry-tagged TALEN plasmids

Two new backbone vectors with EGFP or mCherry tags were created by modifying pCS2TAL3DD (Addgene #37275) and pCS2TAL3RR (Addgene #37276) plasmids, respectively^46^. Using pCS2TAL3DD, pCS2TAL3RR, and pEGFP- and mCherry-carrying plasmids as templates, three fragments were amplified with specific primers (Supplementary Table 1) and the proofreading *pfu* DNA polymerase (Promega, Madison, WI). A restriction site for BsaI was added to both ends of the fragments for subcloning.

The pCS2TAL3DD with EGFP tag and pCS2TAL3RR with mCherry tag were generated by amplifying (I) SP6 promoter with NLS and Flag/HA tag; (II) EGFP/mCherry protein; and (III) Golden Gate RVD assembly system with FokI (DD) domain and SV40 polyA signal. After digesting pCS2TAL3DD and pCS2TAL3RR by HindIII and NotI, and the three amplification products by BsaI, the three fragments were ligated together into the HindIII/NotI sites of pCS2TAL3DD and pCS2TAL3RR respectively, resulting in two new backbone vectors pCS2TAL3-EGFP-DD and pCS2TAL3-mCherry-RR (Supplementary Fig. 2). The tagging of the TALE proteins with fluorescent proteins is for monitoring their expression in the embryos after microinjection with the aim to select the fluorescent embryos with higher mutation rate.

### TALEN target site design and assembly

TALEN target sites on *cyp19a1a* were designed by TAL Effector Nucleotide Targeter (TALE-NT) website (https://tale-nt.cac.cornell.edu/)^45^,^69^. TALEN plasmids were assembled by the Golden Gate TALEN and TAL Effector kit 2.0 according to the protocol published previously in Addgene (Cambridge, MA) with some modifications. The RVD modules were cloned into pCS2TAL3-EGFP-DD and pCS2TAL3-mCherry-RR plasmids to generate the left TALEN and right TALEN respectively. The TALEN site that worked is TGTCTCCTACTGTCGGTTCAT CTGGTCTGGGATCGGGACTGCCAGCAACTACTACAA (spacer sequence underlined).

### CRISPR/Cas9 target site design and single guide RNA (sgRNA) construction

CRISPR/Cas9 target sites were designed using an online tool ZiFiT Targeter software (http://zifit.partners.org/ZiFiT)^70^, which identified sequence 5′GG-(N_18_)-NGG3′ in exon 1 (GGGCTCATAACGGCACTGAAAGG) and exon 2 (GGTTCATCTGGTCTGGGATCGGG) of *cyp19a1a*. Each sgRNA was synthesized by cloning the annealed oligonucleotides into the sgRNA expression vector pDR274 (Addgene #42250) followed by *in vitro* transcription^48^.

### Capped mRNA synthesis

The capped mRNAs for left and right TALEN were generated from NotI-digested pCS2TAL3-EGFP-DD and pCS2TAL3-mCherry-RR plasmids by mMESSAGE mMACHINE SP6 kit (Life Technologies, Grand Island, NY). For CRISPR/Cas9 method, the sgRNAs were from DraI-digested pDR274 sgRNA constructs by MAXIscript T7 kit (Life Technologies). Cas9 RNA was transcribed from pCS2-nCas9n plasmid (Addgene #47929), which is codon-optimized for the zebrafish and contains two nuclear localization signals (NLS) at both the amino and carboxyl termini^71^. pCS2-nCas9n plasmid was linearized by NotI and capped Cas9 mRNA was synthesized by mMESSAGE mMACHINE SP6 kit (Life Technologies). The concentrations of the capped mRNAs were measured by NanoDrop (Thermo Scientific, Waltham, MA) and its quality was examined by agarose gel electrophoresis.

### Microinjection

Microinjection was performed on the zebrafish embryos at one or two-cell stage. A mixture of 300 pg left TALEN and 300 pg right TALEN RNA (4.6 nl) was injected into each embryo. EGFP and mCherry signals were used to confirm if the RNAs were translated in the embryo at 4 h post-fertilization (hpf). Fluorescent signals in TALEN-injected embryos were examined with fluorescent microscope or the Olympus FluoView FV1000 IX81 Confocal Microscope (Olympus, Japan). For CRISPR/Cas9 system, 100 pg sgRNA and 400 pg nCas9n RNA were co-injected. Uninjected embryos were used as controls. All embryos were maintained in an environmental incubator at 28 °C for at least one day before viability examination.

### DNA extraction

Genomic DNA was extracted by incubating each zebrafish embryo or tail fin cut in 50 mM NaOH (50 μl) at 95 °C for 15 min. After cooling to room temperature, one-tenth volume of 1 M Tris (pH8.0) was added to the extract. The solution was centrifuged at top speed for 5 min and the supernatant was used as template for HRMA, HMA or semi-quantitative PCR.

### High resolution melt analysis (HRMA)

Real-time qPCR was performed on the genomic DNA from 20–30 individual embryos/larvae (24 hpf - 15 dpf) or tail fins (20 dpf or older) with SsoFast EvaGreen Supermixes on C1000 Thermal Cycler CFX96 Real-time PCR Detection System (Bio-Rad, Hercules, CA). Primers flanking the target sites were synthesized by IDT (Integrated DNA Technologies, Singapore) (Supplementary Table 1). HRMA was performed at the end of reaction with Precision Melt Analysis software (Bio-Rad) to analyze the difference of the melt curves. For genotyping F2 generation, spiking each sample with WT (+/+) genomic DNA to produce hybrid (+/−) was sometimes necessary to distinguish mutants (−/−) from WT (+/+).

### Conventional PCR and heteroduplex mobility assay (HMA)

Conventional PCR analysis was performed on the genomic DNA using primers listed in Supplementary Table 1. Mutant-specific primer was designed according to the mutated sequence in TALEN-induced Indel mutation. HMA on the same PCR products from HRMA (10–15 individuals) was carried out by native PAGE with 5% for stacking and 20% for separating at a constant voltage of 25 V for 16–18 h. In F1 fish (+/−), homoduplexes and heteroduplexes could be separated with heteroduplexes being slower in mobility^52^. The gel was then stained with ethidium bromide for 15 min before examination.

### DNA sequencing of mutated target sites

Genomic DNA from F1 fish carrying mutations was used for PCR amplification of the target sites (Supplementary Table 1). Ethanol precipitation was used to purify the PCR products for DNA sequencing. The male and female fish with the same frame shift mutations were crossed to produce homozygous F2 mutants (−/−) for phenotype analysis. The F2 fish were further confirmed by DNA sequencing.

### Histological analysis

At least 10 fish were sampled at each time point for each genotype. The entire fish or dissected gonads were freshly fixed in Bouin’s solution followed by dehydration and infiltration. Samples were embedded and processed for paraffin sectioning using microtome (Leica RM2235). Paraffin sections of 5 μm were mounted on slides, deparaffinized, rehydrated and washed with deionized water. The sections were stained with hematoxylin and eosin (H&E), dehydrated, mounted and viewed under the Nikon ECLIPSE Ni-U microscope (Nikon, Japan). All stained sections were captured with Digit Sight DS-Fi2 digital camera (Nikon, Japan). Before sampling for histological analysis, the whole fish was photographed with Canon EOS 7D digital camera and EF 100 mm Macro Lens (Canon, Japan).

### Rescue of mutant phenotype by E2 treatment

The *cyp19a1a* mutant fish were treated with E2 (0.05, 0.50 and 5.00 nM) for 15 days from 15 to 30 dpf. Briefly, the 15-dpf juvenile zebrafish of all three genotypes (+/+, +/−, and −/−) from the same breeding between +/− parents were placed in a 3.5 L tank containing E2. The same volume of stock solvent (350 μL ethanol) was added to the control tank. The water was changed with E2 added every day. The fish were sampled at 35 dpf, 5 days after cessation of the treatment, for genotyping by tail fin cut and fixation in Bouin’s solution for histological observation of gonadal development.

## Additional Information

**How to cite this article**: Lau, E. S.-W. *et al*. Knockout of Zebrafish Ovarian Aromatase Gene (*cyp19a1a*) by TALEN and CRISPR/Cas9 Leads to All-male Offspring Due to Failed Ovarian Differentiation. *Sci. Rep.* **6**, 37357; doi: 10.1038/srep37357 (2016).

**Publisher's note:** Springer Nature remains neutral with regard to jurisdictional claims in published maps and institutional affiliations.

## Supplementary Material

Supplementary Figures and Tables
