# Supplementary material for: Knockout of Zebrafish Ovarian Aromatase Gene (cyp19a1a) by TALEN and CRISPR/Cas9 Leads to All-male Offspring Due to Failed Ovarian Differentiation
Source: Sci Rep. 2016 Nov 23;6:37357. doi: 10.1038/srep37357 (PMC5120357; doi:10.1038/srep37357)
Supplement: Supplementary Figures and Tables [file srep37357-s1.pdf]

Supplementary figures and tables

Knockout of Zebrafish Ovarian Aromatase Gene (*cyp19a1a*) by TALEN and CRISPR/Cas9 Leads to All-male Offspring Due to Failed Ovarian Differentiation

Esther Shuk-Wa LAU[*, 2], Zhiwei ZHANG[*, 1], Mingming QIN[1] and Wei GE[1, 2]

[1] Centre of Reproduction, Development and Aging (CRDA), Faculty of Health Sciences, University of Macau, Taipa, Macau, China; [2]School of Life Sciences, The Chinese University of Hong Kong, Shatin, New Territories, Hong Kong, China

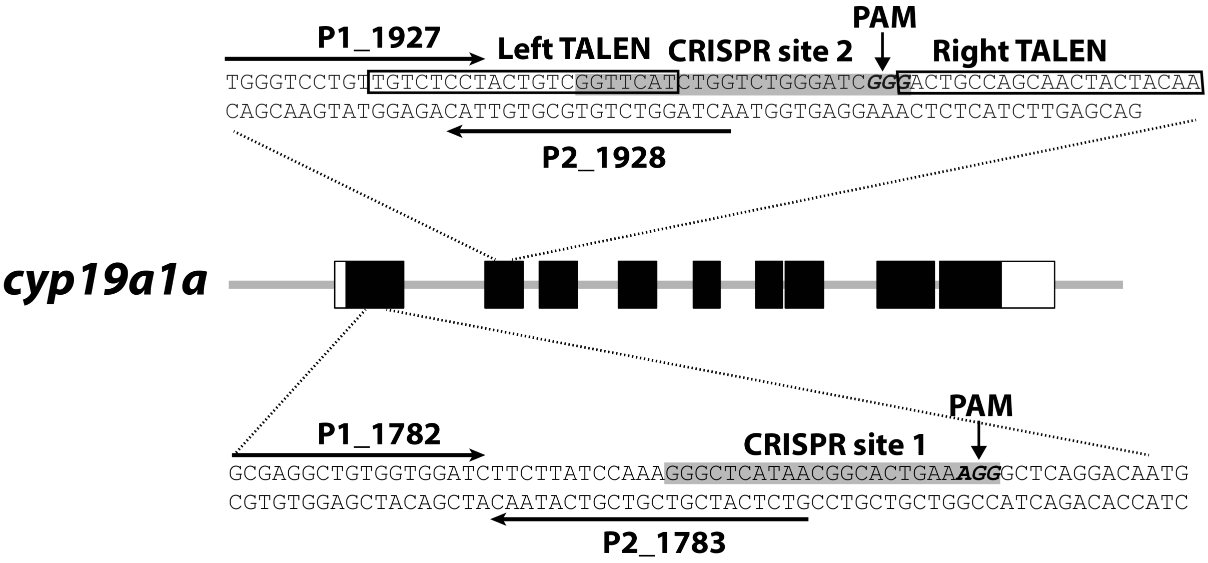

Supplementary Figure 1. Schematic representation of the zebrafish *cyp19a1a* genomic structure and the target sites of TALEN and CRISPR/Cas9. The solid boxes and open boxes represent coding and untranslated exon regions, respectively. The boxed sequences indicate the left and right TALEN binding sites, while the CRISPR sites are shadowed with the PAM sequence italicized. The primers (P1 and P2) used for HRMA and HMA are shown with arrows.

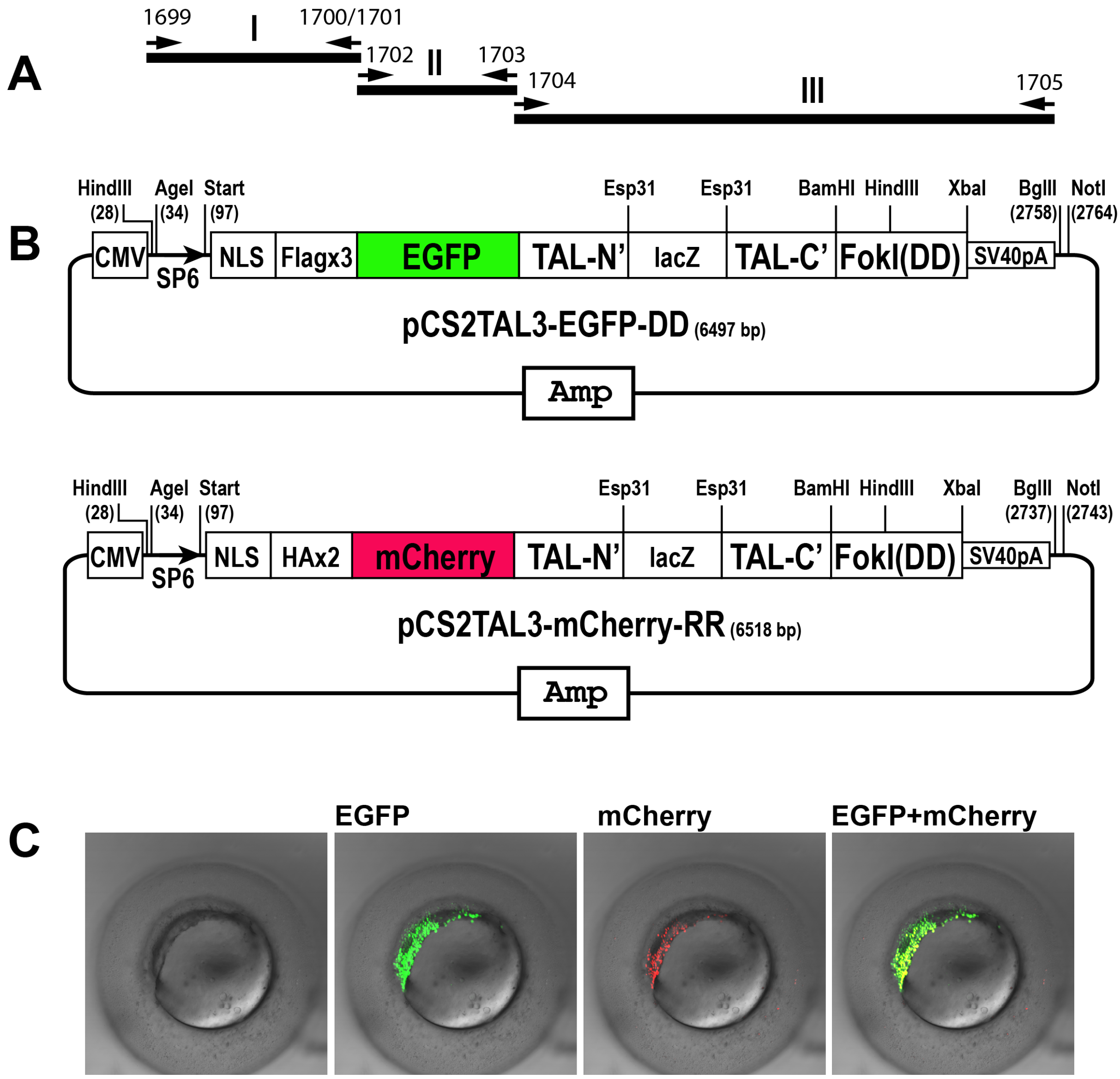

Supplementary Figure 2. Schematic representation of the EGFP and mCherry-tagged TALEN plasmids. A) The assembling strategy to insert EGFP and mCherry genes into the TALEN backbone plasmids. B) Structure of pCS2TAL3-EGFP-DD and pCS2TAL3-mCherry-RR. C). Embryos injected with *in vitro* transcribed mRNA of *cyp19a1a*-pCS2TAL3-EGFP-DD and *cyp19a1a*-pCS2TAL3-mCherry-RR. Both TALE proteins were translated and the yellow signal indicates co-translation of the proteins in the same cells.

Supplementary Table 1 Primers used in this study

**Targets**	**Names**	**Sequences**	**Applications**
pCS2TAL3DD/ pCS2TAL3RR	1699	GGTGCGGTCTC*AAGCTTACCGGT*GATTTAGGTGACACTATAG	Cloning
	1700	GGTGCGGTCTC***GGTACC***CTTGTCATCGTCATCC	
	1701	GGTGCGGTCTC***GGTACC***ATGAGCAGCGTAATCTGG	
	1702	GGTGCGGTCTC***GGTACC***ATGGTGAGCAAGGGCGAGGAG	
	1703	GGTGCGGTCTCTCCACCTTGTACAGCTCGTCCATGCC	
	1704	GGTGCGGTCTCTGTGGATCTACGCACGCTCGGC	
	1705	GTGCGGTCTC**CGGCCG**CAGATCTGAATTAAAAAACCTCCCACAC	
cyp19a1a exon1	1782 (P1)	GCGAGGCTGTGGTGGATC	HRMA, HMA
	1783 (P2)	CAGAGTAGCAGCAGCAGTATTG	
	1755 (P4)	CAAAGGAGCACACAAGGTTACG	DNA sequencing
	1756 (P5)	CAGCAGCAGGCAGAGTAGC	
cyp19a1a exon2	1927 (P1)	TGGGTCCTGTTGTCTCCTAC	HRMA, HMA
	1928 (P2)	TGATCCAGACACGCACAATG	
	1929 (P4)	AACTTTCTGGAAGCCCCAGC	DNA Sequencing
	1930 (P5)	TGATGGTAGATGAACTGTCCCT	
	2001 (P3)	GTTCATCTGGTCTGGGAT	Semi-quantitative PCR
	1930 (P5)	TGATGGTAGATGAACTGTCCCT	
ef1a	728	GGCTGACTGTGCTGTGCTGATTG	Semi-quantitative PCR
	729	CTTGTCGGTGGGACGGCTAGG	

Underlined: BsaI site GGTCTC
Italic: HindIII site AAGCTT
Underlined and italic: AgeI site ACCGGT
Bold and italic: KpnI site GGTACC
Bold: BglII site CGGCCG

Full-length gels (Figure 1F)

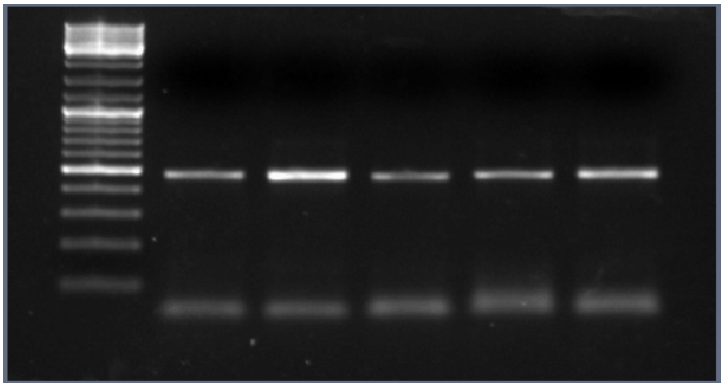

Figure 1F (P4+P5)

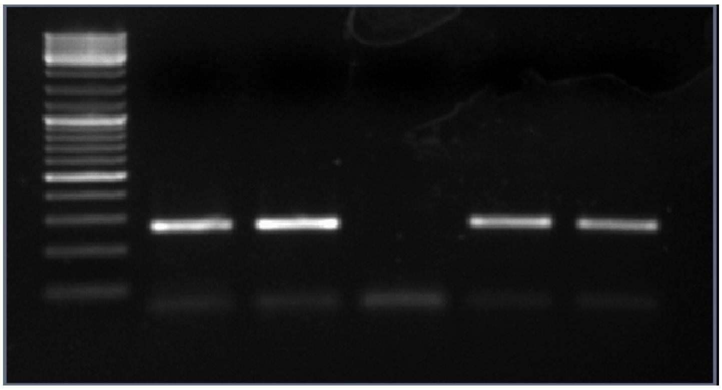

Figure 1F (P3+P5)

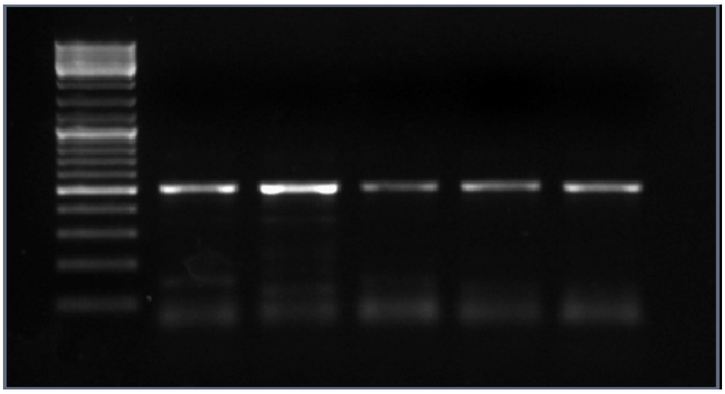

Figure 1F (*ef1a*)
